# Intra-operative tissue diagnosis: isn’t it time for some reporting guidelines?

**DOI:** 10.1007/s00701-016-3057-0

**Published:** 2016-12-23

**Authors:** Walter Stummer

**Affiliations:** Department of Neurosurgery, University of Muenster, Albert-Schweizer Campus 1, 48149 Muenster, Germany

CONSORT, STARD, STROBE, PRISMA .... these acronyms stand for scientific reporting guidelines, all of which are well known to scientifically active clinicians attempting to publish in high-impact medical journals.

Scientist clinicians have long passed the stage where they consider such reporting guidelines more than just a painful detriment to publishing. They have realized that such guidelines are not only important for improving the scientific value and acceptance of their manuscripts. Adhering to such guidelines, even in the early process of designing trials, strongly improves the scientific rigor of their work.

It is little wonder that high-impact journals strictly impose such guidelines, which makes the journals high-impact in the first place, because research published in such journals can be trusted and cited.

In recent years, a new area of research has emerged in neurosurgery: the field of intraoperative tissue imaging or diagnosis. Neurosurgeons have learned that the optical information derived solely from using the surgical microscope is insufficient for identifying relevant disease, and that the surgeon’s perception can be extended by technologies that interrogate tissue for optical information normally not perceived by the neurosurgeon. ALA, introduced by our group, is paradigmatic in this respect. A tumor can be made visible by inducing more or less selective fluorescence in inconspicuous but strongly afflicted brain tissue. This observation and others have spawned a plethora of applications and scientific reports with the aim of finding ways for intra-operative detection of tumor tissue in the brain. Unquestionably, the scientific evaluation of new methods of intra-operative diagnosis must revolve around the interrelation between the diagnostic method and tissue diagnosis. The important question is: Does the fluorescing tissue only represent tumor and is the non-fluorescing tissue normal brain?

One report on intra-operative diagnosis we find in this issue of Acta Neurochirurgica in which the use fluorescein for metastasis is described in 90 patients, as presented by Höhne et al. [2].

The authors illustrate the usefulness of fluorescein by reporting 95% bright fluorescein fluorescence in metastasis, with gross-total resections being achieved in 83% of patients without adverse events being observed. From their observations, the authors conclude their technique to be safe and feasible for increasing the extent of resection in patients with metastasis.

In essence, the paper by Höhne et al. falls into the category of intra-operative tissue diagnosis and the accuracy of such diagnostic imaging. Thus, the appropriate reporting standard would be STARD (**Sta**ndards for **R**eporting of **D**iagnostic Accuracy [1, 3] (http://www.stard-statement.org accessed on December 8, 2016), which was introduced in 1995 in light of a number of concerns surrounding the reporting of studies on diagnostic methods. The Aim of the STARD initiative was to improve the accuracy and completeness of reporting of studies on diagnostic accuracy, allowing readers to assess the potential for bias in a study (internal validity) and to evaluate its generalizability (external validity).

Needless to say, several, if not many, of the 25 requirements formulated in STARD are not clearly reflected by the manuscript by Höhne et al. [2], e.g.,

- to state the research questions or study aims, such as estimating diagnostic accuracy or comparing accuracy between tests or across participant groups,
- regarding data collection: to answer the question of whether data collection were planned before the index test and reference standard were performed (prospective study) or after (retrospective study)?
- to indicate the technical specifications of material and methods involved including how and when measurements were taken, and/or cite references for index tests and reference standard.
- to provide a definition of and a rationale for the units, cut-offs, and/or categories of the results of the index tests and the reference standard.
- to indicate the number, training, and expertise of the persons executing and reading the index tests and the reference standard.

The scientific value of the present report could now be critically discussed, based on its limited adherence to STARD requirements. Basic questions that come to mind might concern the apparently subjective reference standard for efficacy, which is given as “fluorescent staining: ‘bright/helpful’ versus ‘effectively no fluorescence/not helpful’”, or the resection rates on early post-operative MRI of 83% as an indicator of efficacy in this non-randomized, retrospective study.

Critical thoughts concerning the aforementioned paper, however, are not the prime intention of this editorial. Rather, my thoughts revolve along the lines of what would be truly appropriate for reporting diagnostic accuracy in the still-young but expanding field of intraoperative tissue diagnosis.

Even STARD, although helpful, fails to completely account for the special requirements involved in intra-operative tissue diagnosis, because STARD in its original sense merely pertains to laboratory values, such as PSA for prostate cancer, where in respective studies one patient gives a single (plasma) sample.

The particular requirements for intra-operative diagnosis are far more complex: In order to truly determine the diagnostic value of an intra-operative diagnostic method, not one but many samples per patient are required, from both positive and negative areas, possibly from normal brain.

Factors involved in this assessment are (among others) the reporting of the exact location of biopsies, for what reason which location was chosen, how the location was recorded and correlated with imaging (our gold standard), the number of biopsies per location, how multiple biopsies in patients are handled statistically, the timing of biopsies when using tumor markers with a signal time dependency and many other criteria that bias or confound the evaluation of accuracy.

Outcome indicators, such as rates of resection, PFS, or OS require randomization of patients in prospective evaluations to account for selection effects and differences regarding the surgical philosophy in individual centers. True transparency in reporting such optical methods simply requires all the data necessary for the reproduction of results by independent investigators, and for comparing these with the independent investigator’s own, alternate method.

At present, however, no detailed, consented criteria for testing the diagnostic accuracy of intra-operative diagnostic optical imaging are available. Such criteria would allow comparability and reproducibility of methods. The comparative performance of such methods would ultimately be of interest for their future development and application. For this reason, there is a distinct need for clear reporting guidelines focusing on the novel field of intra-operative tissue diagnosis.

I am glad to acknowledge that such reporting guidelines are presently being devised by an international group of neurosurgeons with expertise in intraoperative tissue diagnosis.
